# Prevalence of cardiometabolic diseases in Sub-Saharan Africa: a systematic review and meta-analysis

**DOI:** 10.1080/16549716.2025.2580758

**Published:** 2025-11-13

**Authors:** Shabana Cassambai, John Tetteh, Patrick Highton, Setor K. Kunutsor, Daniel O. Darko, Shavez Jeffers, Deborah Ikhile, George N. Agot, Joyce Olenja, Peter K. Njoroge, Neusa Jessen, Ruksar Abdala, Lauren Senior, Mary A. Amoakoh-Coleman, Kamlesh Khunti, Pamela M. Godia, Alfred E. Yawson, Roberta Lamptey, Kwame O. Buabeng, Albertino Damasceno, Samuel Seidu

**Affiliations:** a Diabetes Research Centre, University of Leicester, Leicester, UK; b Department of Family Medicine, Korle Bu Teaching Hospital, Accra, Ghana; c Division of Musculoskeletal & Dermatological Sciences, The University of Manchester, Manchester, UK; d Section of Cardiology, Department of Internal Medicine, University of Manitoba, Winnipeg, Canada; e Nyaho Medical Centre, Central University, Accra, Ghana; f Department of Physician Assistantship Studies, Central University, Accra, Ghana; g Faculty of Health Sciences, Department of Public and Global Health, University of Nairobi, Nairobi, Kenya; h Faculty of Medicine, Universidade Eduardo Mondlane, Maputo, Mozambique; i Department of Medicine, Maputo Central Hospital, Maputo, Mozambique; j University Hospitals of Leicester, NHS Trust, Leicester, UK; k College of Health Sciences, University of Ghana, Accra, Ghana; l Ghana College of Pharmacists, Accra, Ghana; m School of Pharmacy, University of Allied Health Science, Ho, Ghana

**Keywords:** cardiometabolic disease, type 2 diabetes, hypertension, cardiovascular disease, Sub Saharan Africa

## Abstract

Cardiometabolic diseases (CMDs) are increasingly prevalent in Sub-Saharan Africa (SSA), underscoring the need to understand the existing public health burden. This would guide future policy strategies and interventions to mitigate the challenges posed. The aim of this systematic review was to provide a comprehensive overview of CMDs prevalence in SSA. A PRISMA 2020 compliant systematic literature search was conducted using MEDLINE and The Cochrane Library up to December 2024, including population-based studies with ≥100 participants, aged ≥15 years, and reporting CMDs prevalence in SSA. Random effects meta-analyses were conducted for prevalence, and meta-regression, for temporal trends, evaluated using the median data collection year. Overall, 266 unique studies of 846,511 participants were included; Ethiopia (*n* = 53), Nigeria (*n* = 36) and Ghana (*n* = 20) represented the most studies. Prevalences for the most widely studied condition included type 2 diabetes (T2D) (6.1%; 95% CI = 5.3–7.0), hypertension (27.1%; 95% CI = 25.5–28.8), stroke (1.4%; 95% CI = 1.0–2.0), hypercholesterolemia (11.3%; 95% CI = 7.4–17.0) and cardiovascular diseases (4.8%; 95% CI = 2.5–8.9). The temporal prevalence of hypertension and T2D between 2006 and 2014 showed no statistical significance (β = –0.0289 per year; *p* = 0.11) and (β = 0.0131 per year; *p* = 0.49), respectively. For stroke, a statistically significant temporal decline was observed beginning 2010 (β = b–0.1244 per year; *p* < 0.001). This systematic review reveals a substantial public health burden of CMDs in SSA. The high prevalence emphasises the need for targeted CMDs preventative care strategies in SSA. Notably, most studies were from Ethiopia and Nigeria, indicating the need for more research in other SSA countries for a comprehensive understanding of CMDs in the region.

## Paper context

- **Main findings**: This systematic review highlights the substantial prevalence of cardiometabolic diseases (CMDs) such as hypertension (27.1%) and type 2 diabetes (6.1%) across Sub-Saharan Africa.
- **Added knowledge**: Across 266 studies, the majority of these studies were primarily in Ethiopia, Nigeria, and Ghana, emphasising a need for CMD research in underrepresented countries within the region.
- **Global health impact for policy and action**: This study highlights the need for targeted preventative care strategies to comprehensively address the region’s public health burden.

## Background

The current burden of non-communicable diseases (NCDs) in Sub-Saharan Africa (SSA) is rapidly approaching global prevalence estimates, with prevalence projected to increase by 27% over the next decade [1,2]. Over 1.3 billion people worldwide have hypertension, with more than 31% residing in Low- and Middle-Income Countries (LMICs) [3]. Similarly, 85% of the 852.5 million people living with diabetes worldwide are in LMICs [4]. In SSA, approximately 4–5% of the adult population have type 2 diabetes (T2D), with this figure likely to be higher due to large proportions being unaware [5].

This increase in NCDs, conditions including diabetes, hypertension, hyperlipidaemia, kidney diseases and cardiovascular diseases (CVDs) and are increasingly prevalent in SSA and associated with high morbidity and mortality [6]. The complex reasons for increase in cardiometabolic diseases (CMDs) include (i) increasing urbanisation with consequent negative lifestyle shifts, including reduced physical activity, higher calorie diets, and smoking, (ii) an ageing population, (iii) low health literacy, (iv) wide disparities in accessing self-management education programs among different segments of the population, (v) under funded healthcare systems, and (vi) complex socio-cultural belief systems [7,8].

Unlike communicable diseases, CMDs can be prevented through management of lifestyle factors, including diet, physical activity, and importantly, self-management education [9]. At present, CMDs such as diabetes and hypertension account for approximately 35% of deaths in SSA [10]. Diabetes mellitus and hypertension are the most common CMDs in SSA, with the majority of people being undiagnosed [11,12]. In addition, evidence suggests that CMDs might reduce life-expectancy by 6–10 years [13].

The prevalence estimates for CMDs in SSA are higher in urban areas, compared to rural and economically poorer areas [14]. Therefore, clinics and specialist hospitals are more likely to exist in urban areas. With the lowest levels of awareness, treatment, control of blood pressure worldwide, and increasing average blood pressure readings, there is significant cause for concern in relation to people living with CMDs in SSA [15].

Although there is much data available from country-level studies on CMDs through the WHO Stepwise programme [16,17], there is a need for a comprehensive review to identify the prevalence of CMDs in SSA. The aim of this study was to systematically review evidence on the prevalence of CMDs, such as hypertension, T2D, and CVDs in Sub-Saharan Africa, and describe the prevalence trends over time.

## Methods

### Search strategy and selection criteria

This systematic review and meta-analysis were conducted in accordance with Preferred Reporting Items for Systematic Review and Meta-analyses (PRISMA) guidelines. This review was registered at PROSPERO (CRD42021289258) on 4 November 2021; due to the volume of publications, data on barriers and facilitators to chronic disease care will be reported in a separate review. Databases searched included MEDLINE and The Cochrane Library up to December 2024. While these were the primary sources, the inclusion of regional databases such as African Journals Online (AJOL) further minimises publication bias and improves geographical representation. Example search terms and strategy used for MEDLINE are provided in the supplemental file.

Studies were considered eligible if they included the following: at least 100 people, 15 years and above, from a population-based setting, reported the screening and prevalence of CMDs, and were published after 2015 in order to include the most relevant and contemporary data. We included studies that reported the frequency of at least one of the following: T2D, hypertension, stroke, hypercholesterolaemia, or heart disease. Interventions, serology, histopathology, and clinical biomarkers were beyond the scope of this systematic review. Exclusion criteria included the following: gestational diabetes, pre-hypertension, pre-diabetes, age <15 years old, studies focused on specific cohorts not indicative of the general population, or of groups of people accessing hospital treatment, and articles not in English. Definition of CMDs was by individual studies and we did not exclude based on a predetermined definition.

Titles, abstracts, and full-text articles identified through database searches were evaluated independently by six reviewers to determine whether they met the eligibility criteria using online collaborative software, Rayyan (Massachusetts, USA) [18]. Each article was reviewed by two independent reviewers and disagreements were resolved by consensus or another reviewer. Data extraction was performed using a pre-specified and piloted data extraction form and double checked by a second reviewer. Fields included study details, population demographics, relevant outcomes, hypertension, and diabetes cut-off and guidance.

### Data analysis

Prevalence estimates with 95% confidence intervals were pooled across studies using the updated – metan – command with a logit transformation [19]. Random-effects models by DerSimonian and Laird, which takes into account heterogeneity both within and between studies, were used to pool prevalence estimates to account for the effect of heterogeneity [20]. Between study statistical heterogeneity was quantified using standard chi-square tests and the I^2^ statistic [21]. The I^2^ values were interpreted by grouping these values into various heterogeneity categories, in accordance with the *Cochrane Handbook* guidelines: 0–40% (‘low’), 30–60% (‘moderate’), 50–90% (‘high/substantial’), and 75–100% (‘very high/considerable’) heterogeneity [22]. Funnel plots and the Egger’s regression symmetry test were used to assess for publication bias for outcomes with ≥10 studies [23]. Pre-specified study-level characteristics such as geographical sub-region (Central vs Eastern vs Northern vs Southern vs Western), study setting (Rural vs Urban vs Mixed vs Unknown), average age at baseline (≥50 vs <50 years), and risk of bias scores (Good vs Fair vs Poor) were explored as sources of heterogeneity, using stratified analysis and random effects meta-regression [24]. Temporal trends in prevalence were evaluated using the median year of data collection reported by studies, consistent with previous reports [25,26]. When a study reported a range of years for data collection (e.g. 1985–1990), the median year of the range was calculated and assigned to that study. For studies that reported only a single year of data collection, that year was used directly. We did not use publication year to derive temporal trends. We also conducted random-effects meta-regression analyses, with the prevalence estimate as the dependent variable and the year of data collection as the independent variable. Regression coefficients (β) from the meta-regression represent the average annual change in prevalence (% per year), and we present the β coefficient together with its 95% confidence interval (CI) and *p*-value to assess statistical significance. All statistical analyses were conducted using STATA 18 (Stata Corp, College Station, Texas, USA).

### Study quality assessment

Two reviewers independently assessed the quality of the included studies. The study quality assessment tools of NIH for quality assessment of observational cohort and cross-sectional studies and case series studies [27] were used to assess the quality of included studies. An overall quality rating of good, fair, or poor was determined for each study.

## Results

### Characteristics of included studies and populations

**Figure 1.**
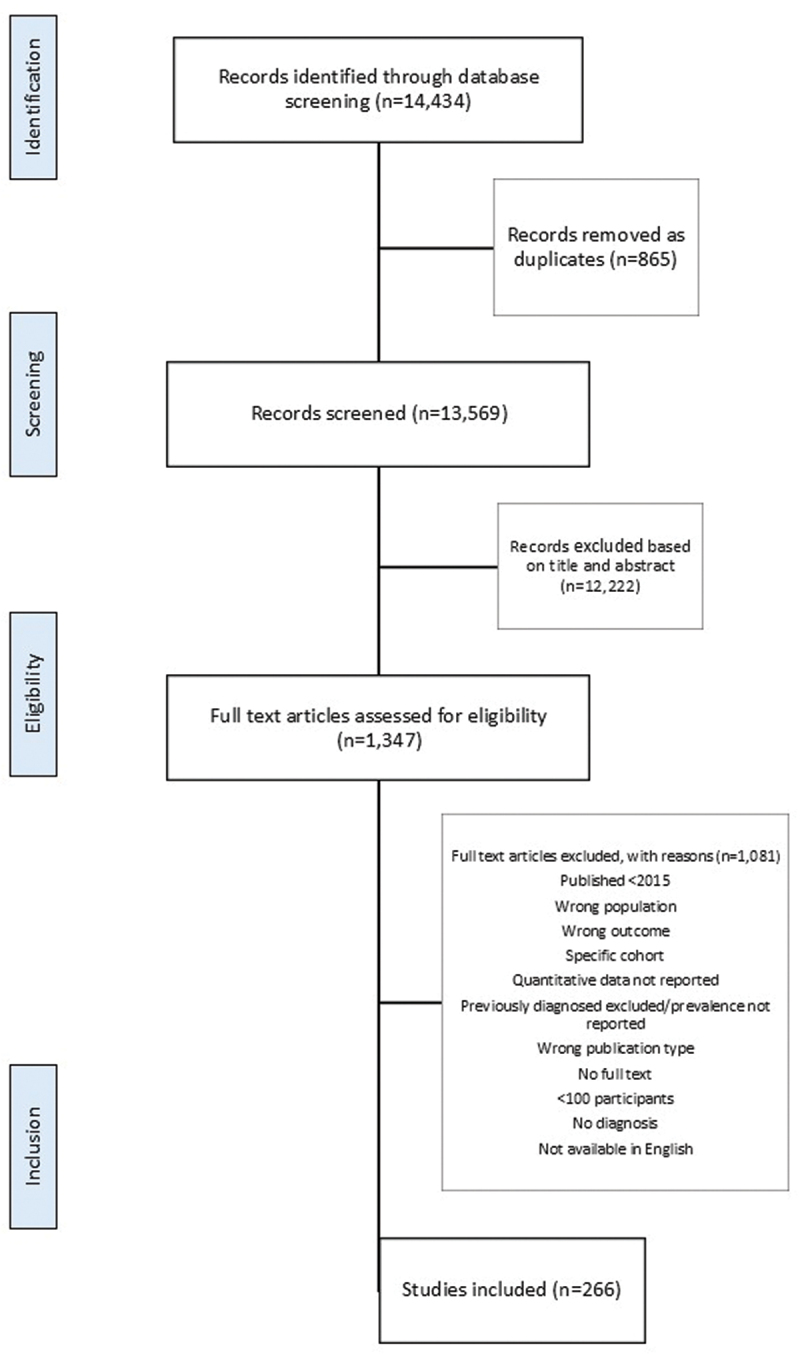
PRISMA flowchart.

A total of 13,965 titles were identified through database searches of which 266 titles were included in this review (Figure 1). Studies were published between 2015 and 2025, with data collection years ranging from 2006 to 2023. In total, a total of 846,511 participants were included in this analysis. Table 1 shows the baseline characteristics and geographical split of studies according to sub-regions of SSA, defined as Eastern, Northern, Western, Southern, and Central. Study settings were reported as urban, rural, mixed, or unknown. The mean age range and weighted mean age were 21.7–71.6; 41.0 years for hypertension; 31.0–64.0; 40.4 years for T2D; 32.5–44.0; 34.2 years for hypercholesterolemia; 36.7–55.1; 41.6 years for CVD; and 35.1–64.0; 44.2 years for stroke.

**Table 1.** Baseline characteristics and geographical distribution of studies as per hypertension, type 2 diabetes, hypercholesterolaemia, cardiovascular disease, and stroke.

			Regions (no. of studies)					ROB (no. of studies)		
No. of studies	Mean age range, yrs	Weighted mean age, yrs	Central	Eastern	Northern	Southern	Western	Good	Fair	Poor
Hypertension										
229	21.7–71.6	41.0	20	87	9	25	88	124	91	14
Type 2 diabetes										
116	31.0–64.0	40.4	12	37	8	17	42	65	44	7
Hypercholesterolemia										
8	32.5–44.0	34.2	2	2	1	0	3	3	5	–
Cardiovascular disease										
8	36.7–55.1	41.6	0	5	0	0	3	5	3	–
Stroke										
17	35.1–64.0	44.2	3	2	1	5	6	6	10	1

Due to overlapping participants, the total number of studies does not add up to the total number of unique studies (278). ROB, risk of bias.

### Definitions of hypertension and type 2 diabetes

Hypertension and diabetes were defined according to the reported guidelines within each study. For hypertension, the European Society of Cardiology/European Society of Hypertension (ESC/ESH), American College of Cardiology/American Heart Association (ACC/AHA) [28], WHO STEPS [16], WHO SAGE [29], Joint National Committee report 7/8 (JNC7/8) [30], American Society of Hypertension/International Society of Hypertension (ASH/ISH) [31], National Cholesterol Education Program (NCEP) [32], British Society for Haematology Standard (BSHS) [33], Health and Retirement Study (HRS) 2010 [34] or local guidelines were used as a cut off. Of these reported guidelines, the majority of studies defined hypertension as blood pressure above 140 mmHg systolic, and 90 mmHg diastolic, a few studies used a systolic pressure of 120–135 mmHg (*n* = 8) or 150+ (*n* = 5) or did not report the guidelines followed, there was no information collated for where blood pressure was measured. In the case of T2D, the International Diabetes Federation (IDF) [35], American Diabetes Association (ADA) [36], WHO/WHO STEPS [16], or National NCDs guidelines were referenced to report the diabetes cut off. The guidelines either reported on HbA1c, fasting glucose or random blood glucose values, these were defined as >126 mg/dL or >7.0 mmol/L for fasting plasma glucose, >6.1 mmol/L for fasting blood glucose, ≥6.5% for HbA1c, >200 mg/dL, or >11 mmol/L for random blood glucose.

### Pooled prevalence estimates

**Figure 2.**
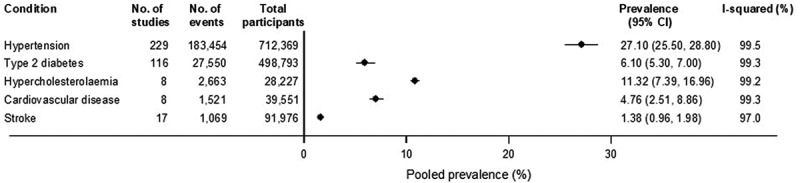
Summary plot of prevalence estimates of evaluated conditions.

The summary plot for the pooled prevalence estimates of hypertension, T2D hypercholesterolaemia, CVD, and stroke is presented in Figure 2.

**Figure 3.**
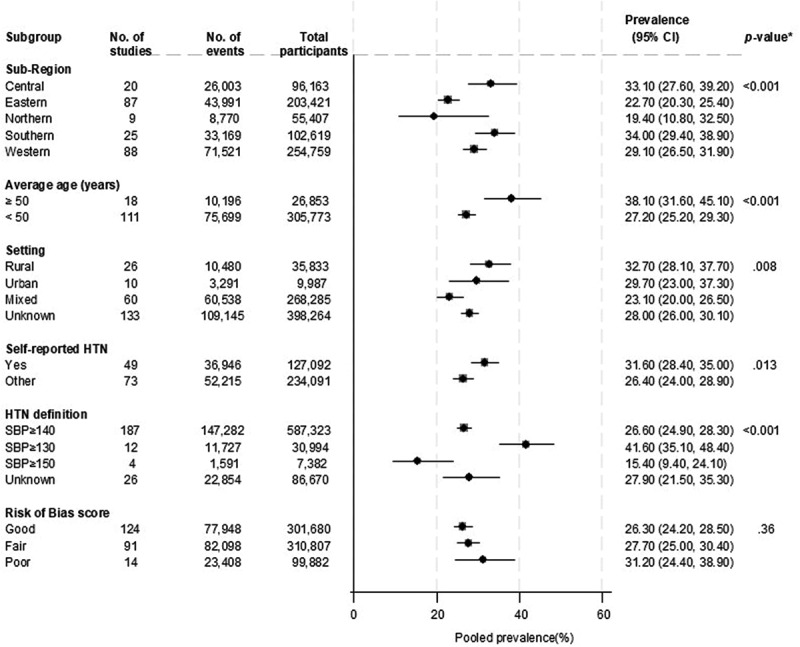
Prevalence of hypertension according to study level characteristics.

**Hypertension** - Across 229 studies, hypertension prevalence ranged widely from 0.2% to 72.8%, reflecting significant heterogeneity across regions, methodologies, and population demographics (Supplemental file 2; Figure S1). The pooled estimate was 27.1% (95% CI: 25.5–28.8, *I*^2^ = 99.5%). The mean age across studies was 41.9 years (8.1 SD), with 43.5% males. Ten studies reported on urban populations, whereas 26 reported on rural populations, the rest were mixed settings or unknown (Supplemental file 2). There was evidence of substantial heterogeneity between studies, which could partly be explained by study characteristics such as study region (*p* for meta-regression < .001), mean age (*p* for meta-regression <.001), whether hypertension was self-reported (*p* for meta-regression = .013), definition of hypertension (*p* for meta-regression <.001) and risk of bias score (*p* for meta-regression .036) (Figure 3). The pooled prevalence of hypertension was higher in South Africa (34.0%) and Central Africa (33.1%) compared to other regions. People who were aged ≥50 years had a higher prevalence of hypertension compared with individuals <50 years (38.1% vs. 27.2%).

*P-values for heterogeneity.

The prevalence of hypertension was higher in individuals who self-reported hypertension than those who did not (31.6% vs. 26.4%). The prevalence of hypertension was higher (41.6%) when a systolic blood pressure cut-off of ≥130 mmHg was used to define hypertension compared with other definitions. Studies with a poor risk of bias score reported higher hypertension prevalence (31.2%) than studies with good and fair scores (26.3% and 27.7%, respectively) (Supplemental file 2).

**Figure 4.**
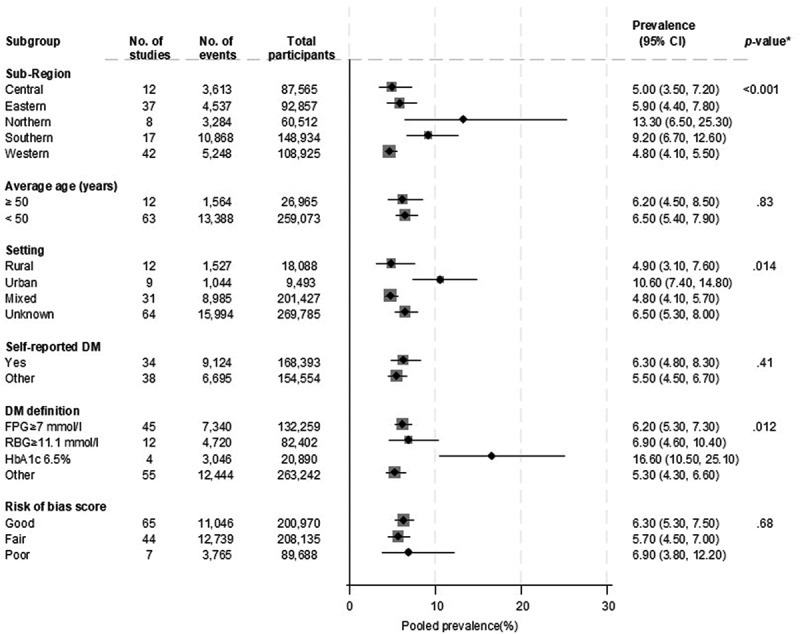
Prevalence of type 2 diabetes according to study level characteristics.

**T2D** - Across 116 studies, the reported prevalence of T2D ranged from 0.3% to 63.9%, with a pooled prevalence of 6.1% (5.3–7.0, *I*^2^ = 99.3%). The mean age across studies was 43.2 years (8.0 SD), with 43.3% males (Supplemental file 2; Figure S2). There were 9 studies in an urban setting, whereas 12 reported on rural populations, the rest were mixed or unknown (Supplemental file 2). There was evidence of substantial heterogeneity between studies, which could partly be explained by the study region (*p* for meta-regression < .001), study setting (*p* for meta-regression = .014), and definition of T2D (*p* for meta-regression = .012). Higher prevalence estimates were recorded for North Africa (13.3%) than other regions, urban settings (10.6%) than other settings, and T2D defined using HbA1c (16.6%) than other definitions for T2D (Figure 4).

*P-values for heterogeneity.

**Hypercholesterolaemia** - Across eight studies, the prevalence of hypercholesterolemia ranged from 4.0% to 29.8%, with a pooled prevalence of 11.3% (7.4–17.0, *I*^2^ = 99.2%) (Figure 2). These studies presented a mean population age of 38.3 years (5.8 SD) and 40.9% male, with 1 study in a rural setting and the others with mixed or unknown settings (Supplemental file 2; Figure S3).

**CVD** - Eight studies also reported on CVD with prevalence estimates ranging from 0.7% to 25.3%, with a pooled prevalence of 4.8% (2.5–8.9, *I*^2^ = 99.3%) (Figure 2). The mean age across the studies was 49.2 years (5.8 SD), with 44.3% males and two studies based in a rural setting (Supplemental file 2; Figure S4).

**Figure 5.**
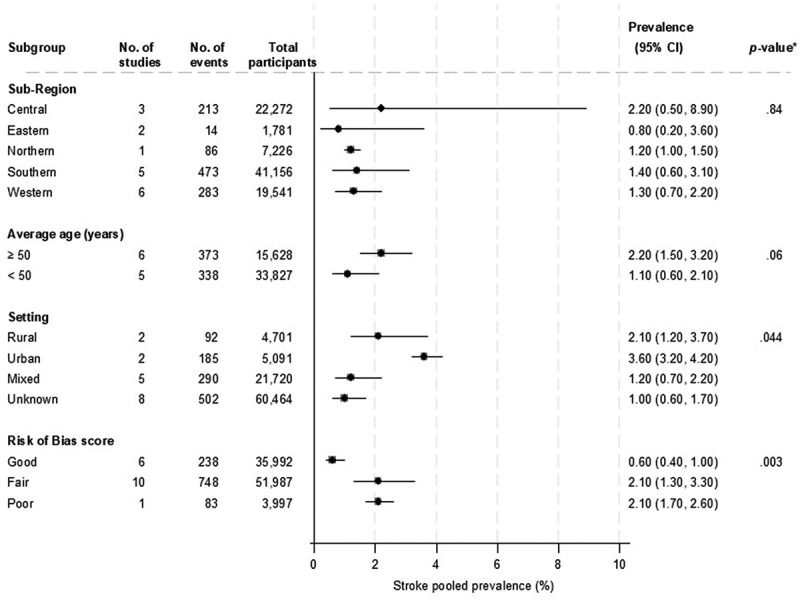
Prevalence of stroke according to study level characteristics.

**Stroke** - Across 17 studies, the prevalence of stroke ranged from 0.2% to 7.3%, with a pooled prevalence of 1.4% (1.0–2.0, *I*^2^ = 97.0%). The mean age across studies was 50.0 years (10.7 SD), with 45.6% males (Supplemental file 2; Figure S5). Two studies each were in urban and rural settings, and the rest were mixed or unknown. There was evidence of substantial heterogeneity between studies, which could partly be explained by the study setting (*p* for meta-regression = .044) and risk of bias score (*p* for meta-regression = .003). Higher prevalence estimates were recorded for urban settings (3.6%) than other settings, and studies with poor or fair risk of bias scores (2.1% each) than studies with good scores (0.6%) (Figure 5).

*P-values for heterogeneity.

### Publication bias

**Figure 6.**
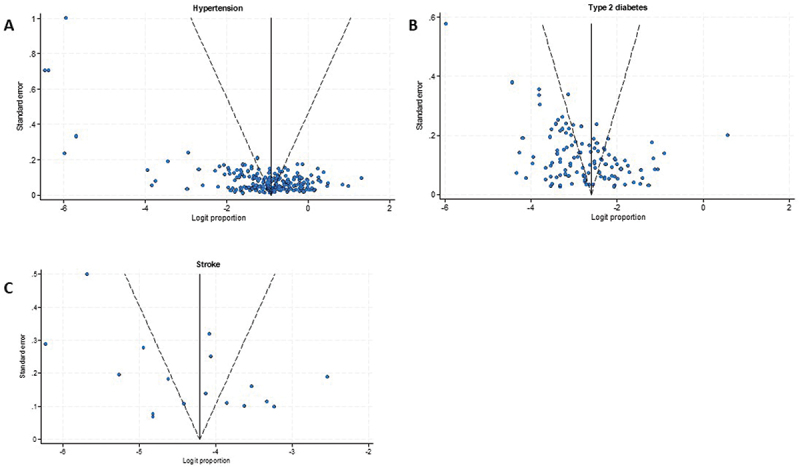
Funnel plots for studies on hypertension (a), type 2 diabetes (b), and stroke (c).

Funnel plots for studies on hypertension, T2D, and stroke are presented in Figure 6. Egger’s regression tests showed no significant evidence of publication bias for any outcome: hypertension (*p* = 0.57), T2D (*p* = 0.44), and stroke (*p* = 0.87).

**Figure 7.**
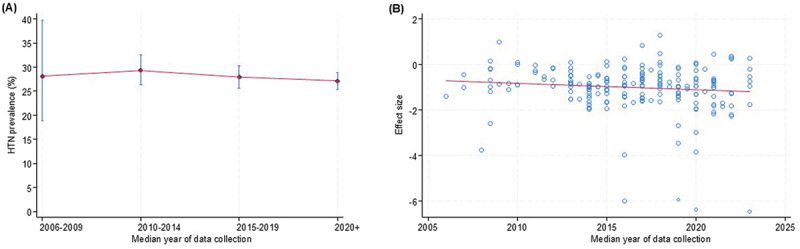
Temporal trends in prevalence of hypertension*: (a) prevalence of hypertension by median year of data collection; (b) meta-regression bubble plot of prevalence of hypertension against median year of study data collection.

**Figure 8.**
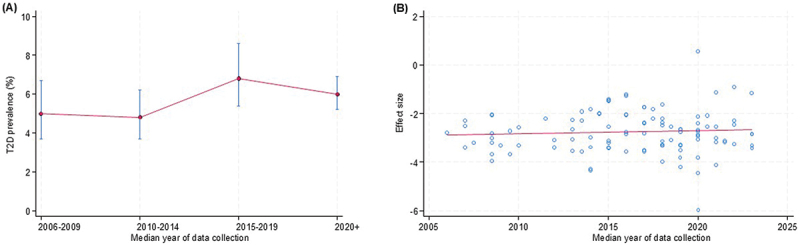
Temporal trends in prevalence of type 2 diabetes*: (a) prevalence of type 2 diabetes by median year of data collection; (b) meta-regression bubble plot of prevalence of type 2 diabetes against median year of study data collection.

**Figure 9.**
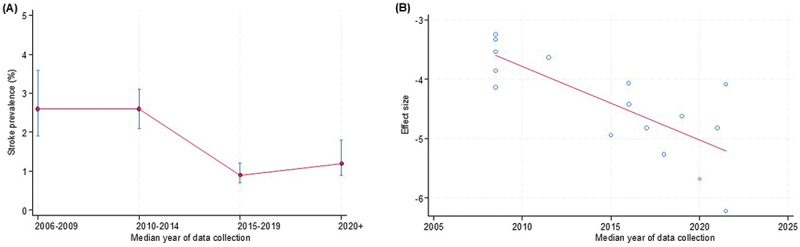
Temporal trends in prevalence of stroke*: (a) prevalence of stroke by median year of data collection; (b) meta-regression bubble plot of prevalence of stroke against median year of study data collection.

Trends in prevalence (Figures 7–9) show the temporal trends in prevalence of hypertension, T2D, and stroke by median year of data collection. For hypertension, prevalence remained relatively stable between 2006 and 2014, followed by a gradual decline from 2015 onwards (Figure 7a). In meta-regression analyses, no statistically significant association was observed between hypertension prevalence and year of data collection (β = −0.0289 per year; 95% CI: −0.0642 to 0.0065; *p* = 0.11; τ^2^ = 0.858) (Figure 7b).

*capped vertical bars represent 95% confidence intervals.

*capped vertical bars represent 95% confidence intervals.

*capped vertical bars represent 95% confidence intervals.

For T2D, prevalence was largely stable between 2006 and 2014, showed a modest increase thereafter, and subsequently declined from 2019 onwards (Figure 8a). Meta-regression analysis did not demonstrate a statistically significant temporal trend (β = 0.0131 per year; 95% CI: −0.0247 to 0.0509; *p* = 0.49; τ^2^ = 0.713) (Figure 8b).

For stroke, prevalence remained constant between 2006 and 2014, declined sharply in subsequent years, and then showed a slight increase from 2019 onwards (Figure 9a). Meta-regression analyses provided statistically significant evidence of a temporal decline beginning around 2010 (β = −0.1244 per year; 95% CI: −0.1802 to −0.0686; *p* < 0.001; τ^2^ = 0.203) (Figure 9b).

## Discussion

This contemporary systematic review reports data on the prevalence of 27.1% for hypertension, 6.1% for T2D, 11.3% for hypercholesterolaemia, 1.4% for stroke, and 4.8% for CVDs for adolescents and adults (>15 years age) in SSA. However, other systematic reviews show a much higher prevalence of over 45% for hypertension alone [37–39]. Although these studies report a prevalence which is double that reported in this review, these reviews were published between 2017 and 2019, included a smaller number of studies, and selected hospitalised populations e.g. hospital/clinic-based studies; these populations would most likely include more severe cases and therefore prevalence rates would be expected to be higher. The findings of our systematic review are more in line with data reported from WHO STEPS surveys, which reported a pooled prevalence of 24.9% amongst nine countries between 2014 and 2017 [40]. Central and South African populations had the highest prevalence of hypertension, with overall prevalence particularly higher in individuals aged over 50 years. This correlates with findings from the WHO STEPS survey, widely implemented in many SSA countries, showing a high hypertension prevalence in countries such as Cameroon, Botswana, Lesotho, Eswatini [41,42], and Mozambique [43]. Although previous studies have reported higher rates of hypertension in urban settings [44], our study reports no significant difference for urban and rural areas. This is more in line with recent studies which have shown an increase in the prevalence of hypertension in rural areas due to increased urbanisation, which has led to adoption of negative lifestyle choices [45]. It should be noted that of the 229 studies that assessed hypertension, only 26 were in a rural setting, and 10 in an urban setting. The majority of studies included in our review either present mixed populations (*n* = 60) or do not report on setting (*n* = 133) and therefore this is likely to affect the overall result.

Our findings demonstrated a pooled prevalence of T2D of 6.1% in the adolescent and adult population of SSA, with a higher prevalence in urban areas (10.6%). This is slightly higher than the recent International Diabetes Federation (IDF) report, which suggests a prevalence of 5.0% in adults aged 20–79 years in the same region [4]. The difference in our reporting may be due to inclusion of individuals aged 15 years and over in this study. As more people migrate to cities for jobs, this could lead to increased T2D prevalence, necessitating enhanced urban healthcare infrastructure. Considering the higher burden of undiagnosed diabetes in SSA [46], the total prevalence of T2D is likely to be much higher, with the IDF report suggesting that 72.6% adult living with T2D in SSA are undiagnosed [4]. The 5.0% age standardised prevalence in type 2 diabetes by IDF is higher than the previously reported 4.5% prevalence in 2021 [47], this confirms the rise in prevalence within this region, with an expected increase of 142% by 2050 [4]. We observed a higher prevalence when T2D diagnosis was made using HbA1c rather than an oral glucose tolerance test. HbA1c levels are generally elevated in Black populations and show a strong correlation with retinopathy when they surpass 6.5% [48]

In addition to the prevalence reported for hypertension and T2D, we also report prevalence on hypercholesterolaemia, CVDs, and stroke. Previous systematic reviews within the last 5 years reported on risk factors for these conditions or focused on a singular condition; this review provides a comprehensive overview of CMDs prevalence in the region. Our findings show a lower prevalence of hypercholesterolaemia in comparison to other studies [49,50]; however, these studies were either restricted to specialised populations, i.e. hospitalised patients or those attending clinic, or were only representative of a single country. Our systematic review also reports a lower prevalence for CVDs and stroke compared to other studies. The prevalence of stroke and CVDs in SSA is reported to vary across different countries and in different settings [51,52], this may in part explain the discrepancies with the current study. Particularly in the case of stroke, the reporting norms for prevalence are cases for 100,000 people, our study shows a prevalence of 1,400 per 100,000 individuals; this is slightly higher than the reported age-standardised prevalence of 981 per 100,000 [53]. However, the prevalence of stroke is thought to vary between countries in SSA and can range from 15 to 1460 per 100,000 persons [54]. Stroke-related mortality in SSA is also very high when compared to high-income countries, due to a higher percentage of haemorrhagic stroke; this may also explain a lower prevalence reported for stroke overall in SSA. It is also of note that the number of studies and cases presented in this review are low and may therefore contribute to the differences reported for CVDs and hypercholesterolaemia prevalence compared to the literature.

This systematic review intended to highlight the prevalence of CMDs, notably hypertension and T2D, in SSA, stratified by region, setting, age, disease definition, diagnostic guidelines, and self-reported diagnosis. Furthermore, very few studies reported data on ethnic or tribal groups or socioeconomic status and therefore data could not be stratified by these. Interestingly, our analyses show that in the case of hypertension, there is no significant difference in reported prevalence for rural, urban, or mixed populations. This contrasts with other studies which show that individuals in urban populations show a higher prevalence of hypertension than those in rural populations [44,55]. This may be due to increasing availability of processed foods in rural areas [7]; in more recent years, the roll out of NCDs surveillance programmes such as WHO STEPwise has looked to improve health surveillance and monitoring in rural and urban parts of SSA [56], thus providing comparable prevalence data for otherwise less accessed areas.

## Strengths and limitations

A large number of studies included in this review included self-reported hypertension and T2D, which limits the understanding of diagnostic measures used in the population context. Similarly, the use of various international guidelines for defining hypertension showed differences in diagnosis and may also have an effect on treatment guidance. The values used by a lot of guidelines often vary depending on where it was measured, e.g. at home vs in clinic, which may result in underestimation of prevalence, particularly in areas where higher systolic blood pressure is used as a cut-off. The analysis of this study also does not report on prevalence in males and females, but rather reports prevalence as a combination of these groups, this may also impact the overall prevalence presented, as other studies have previously reported that male populations present with a higher prevalence of cardiometabolic disease [44]. Only one study [42] reported in this review reports on median age <18; however, this study includes data from multiple countries on hypertension prevalence (Supplementary file 2). Although all other studies report a median or mean age of above 18, not all studies report prevalence reported by age range and therefore we have only reported pooled population prevalence. Whilst the studies included within this review are representative of the regions of SSA, there is a skew in the number of studies reported in individual countries, such that Ethiopia and Nigeria largely dominate the representation of the East and West African regions, respectively. This means that although there is a large representation of East and West African countries, the majority of the data is from the two aforementioned countries, although this may not reflect the prevalence in other countries within the region, it is important to recognise that the countries represent two of the most populated countries in SSA.

This review also draws attention to hypercholesterolaemia and CVDs in SSA; however, the number of studies representatives of these conditions is low (*n* = 8, each respectively) and therefore warrants investigation in larger national studies. There is also the potential for publication bias, as this review primarily includes studies published in accessible databases. This might exclude relevant data from unpublished studies or those in less accessible local journals, leading to an incomplete assessment of CMDs prevalence in SSA.

## Conclusion

This systematic review provides a comprehensive overview of the burden of CMDs in SSA. The prevalence of hypertension is most dominant across all geographical regions, with Central and South African regions showing the highest prevalence. With T2D, the increased prevalence is most apparent in the North African region, particularly in urban settings. Prevalence is high in younger groups, with higher risk of adverse events at a younger age; this may have major consequences on economic productivity for the region. Generally, this study highlights the rise in prevalence of CMDs in SSA and emphasises the need for early, comprehensive intervention to ensure better preventative strategies, detection, and effective management of the cardiometabolic diseases in these populations.

## Supplementary Material

CREATE_Supplementary 1.docx

PRISMA_2020_checklist_CREATE SR.docx

Supplemental 2_CREATE_ALL data.xlsx

## Data Availability

The authors confirm that the data supporting the findings of this study are available within the article and its supplementary materials.
