# Supplementary material for: Prevalence of cardiometabolic diseases in Sub-Saharan Africa: a systematic review and meta-analysis
Source: Glob Health Action. 2025 Nov 13;18(1):2580758. doi: 10.1080/16549716.2025.2580758 (PMC12616651; doi:10.1080/16549716.2025.2580758)
Supplement: CREATE_Supplementary 1.docx [file ZGHA_A_2580758_SM0245.docx]

# Supplementary File 1

## Supplementary 1: Ovid MEDLINE(R) ALL <1946 to January 22, 2024>

1 "Africa South of the Sahara"/ or Africa/ 44642

2 ("Africa south of the Sahara" or "sub$saharan Africa").mp. [mp=title, book title, abstract, original title, name of substance word, subject heading word, floating sub-heading word, keyword heading word, organism supplementary concept word, protocol supplementary concept word, rare disease supplementary concept word, unique identifier, synonyms, population supplementary concept word, anatomy supplementary concept word] 17839

3 1 or 2 48258

4 angola.mp. or Angola/ 2120

5 Benin/ 2035

6 (benin or dahomey).mp. [mp=title, book title, abstract, original title, name of substance word, subject heading word, floating sub-heading word, keyword heading word, organism supplementary concept word, protocol supplementary concept word, rare disease supplementary concept word, unique identifier, synonyms, population supplementary concept word, anatomy supplementary concept word] 5089

7 5 or 6 5089

8 Botswana/ 2288

9 (botswana or bechuanaland or kalahari).mp. [mp=title, book title, abstract, original title, name of substance word, subject heading word, floating sub-heading word, keyword heading word, organism supplementary concept word, protocol supplementary concept word, rare disease supplementary concept word, unique identifier, synonyms, population supplementary concept word, anatomy supplementary concept word] 3766

10 8 or 9 3766

11 Burkina Faso/ 4221

12 ("burkina faso" or "burkina fasso" or "upper volta").mp. [mp=title, book title, abstract, original title, name of substance word, subject heading word, floating sub-heading word, keyword heading word, organism supplementary concept word, protocol supplementary concept word, rare disease supplementary concept word, unique identifier, synonyms, population supplementary concept word, anatomy supplementary concept word] 6257

13 11 or 12 6257

14 burundi.mp. or Burundi/ 1304

15 Cabo Verde/ 273

16 ("cabo verde" or "cape verde").mp. [mp=title, book title, abstract, original title, name of substance word, subject heading word, floating sub-heading word, keyword heading word, organism supplementary concept word, protocol supplementary concept word, rare disease supplementary concept word, unique identifier, synonyms, population supplementary concept word, anatomy supplementary concept word] 885

17 15 or 16 885

18 cameroon.mp. or Cameroon/ 10495

19 Central African Republic/ 863

20 ("central african republic" or ubangi-shari).mp. [mp=title, book title, abstract, original title, name of substance word, subject heading word, floating sub-heading word, keyword heading word, organism supplementary concept word, protocol supplementary concept word, rare disease supplementary concept word, unique identifier, synonyms, population supplementary concept word, anatomy supplementary concept word] 1439

21 19 or 20 1439

22 chad.mp. or Chad/ 1798

23 Comoros/ 391

24 (comoro* or comores or mayotte).mp. [mp=title, book title, abstract, original title, name of substance word, subject heading word, floating sub-heading word, keyword heading word, organism supplementary concept word, protocol supplementary concept word, rare disease supplementary concept word, unique identifier, synonyms, population supplementary concept word, anatomy supplementary concept word] 920

25 23 or 24 920

26 "Democratic Republic of the Congo"/ 5325

27 ("belgian congo" or congo or kinshasa or "democratic republic of the congo" or katanga or zaire).mp. [mp=title, book title, abstract, original title, name of substance word, subject heading word, floating sub-heading word, keyword heading word, organism supplementary concept word, protocol supplementary concept word, rare disease supplementary concept word, unique identifier, synonyms, population supplementary concept word, anatomy supplementary concept word] 22041

28 26 or 27 22041

29 Congo/ 2058

30 (congo or brazzaville or "republic of the congo").mp. [mp=title, book title, abstract, original title, name of substance word, subject heading word, floating sub-heading word, keyword heading word, organism supplementary concept word, protocol supplementary concept word, rare disease supplementary concept word, unique identifier, synonyms, population supplementary concept word, anatomy supplementary concept word] 20892

31 29 or 30 20892

32 Cote d'Ivoire/ 3702

33 ("cote d'ivoire" or "ivory coast" or "cote diivoire").mp. [mp=title, book title, abstract, original title, name of substance word, subject heading word, floating sub-heading word, keyword heading word, organism supplementary concept word, protocol supplementary concept word, rare disease supplementary concept word, unique identifier, synonyms, population supplementary concept word, anatomy supplementary concept word] 5554

34 32 or 33 5554

35 Equatorial Guinea/ 334

36 ("equatorial guinea" or "spanish guinea" or "rio muni").mp. [mp=title, book title, abstract, original title, name of substance word, subject heading word, floating sub-heading word, keyword heading word, organism supplementary concept word, protocol supplementary concept word, rare disease supplementary concept word, unique identifier, synonyms, population supplementary concept word, anatomy supplementary concept word] 632

37 35 or 36 632

38 eritrea.mp. or Eritrea/ 887

39 Eswatini/ 804

40 (eswatini or swaziland).mp. [mp=title, book title, abstract, original title, name of substance word, subject heading word, floating sub-heading word, keyword heading word, organism supplementary concept word, protocol supplementary concept word, rare disease supplementary concept word, unique identifier, synonyms, population supplementary concept word, anatomy supplementary concept word] 1364

41 39 or 40 1364

42 ethiopia.mp. or Ethiopia/ 35207

43 Gabon/ 1697

44 (gabon or "gabonese republic").mp. [mp=title, book title, abstract, original title, name of substance word, subject heading word, floating sub-heading word, keyword heading word, organism supplementary concept word, protocol supplementary concept word, rare disease supplementary concept word, unique identifier, synonyms, population supplementary concept word, anatomy supplementary concept word] 2584

45 43 or 44 2584

46 gambia.mp. or Gambia/ 3856

47 Ghana/ 12289

48 (ghana or "gold coast").mp. [mp=title, book title, abstract, original title, name of substance word, subject heading word, floating sub-heading word, keyword heading word, organism supplementary concept word, protocol supplementary concept word, rare disease supplementary concept word, unique identifier, synonyms, population supplementary concept word, anatomy supplementary concept word] 19072

49 47 or 48 19072

50 guinea.mp. or Guinea/ 167261

51 guinea-bissau.mp. or Guinea-Bissau/ 1462

52 kenya.mp. or Kenya/ 29258

53 Lesotho/ 578

54 (lesotho or basutoland).mp. [mp=title, book title, abstract, original title, name of substance word, subject heading word, floating sub-heading word, keyword heading word, organism supplementary concept word, protocol supplementary concept word, rare disease supplementary concept word, unique identifier, synonyms, population supplementary concept word, anatomy supplementary concept word] 1135

55 53 or 54 1135

56 liberia.mp. or Liberia/ 2420

57 Madagascar/ 4052

58 (madagascar or "malagasy republic").mp. [mp=title, book title, abstract, original title, name of substance word, subject heading word, floating sub-heading word, keyword heading word, organism supplementary concept word, protocol supplementary concept word, rare disease supplementary concept word, unique identifier, synonyms, population supplementary concept word, anatomy supplementary concept word] 6750

59 57 or 58 6750

60 Malawi/ 7138

61 (malawi or nyasaland).mp. [mp=title, book title, abstract, original title, name of substance word, subject heading word, floating sub-heading word, keyword heading word, organism supplementary concept word, protocol supplementary concept word, rare disease supplementary concept word, unique identifier, synonyms, population supplementary concept word, anatomy supplementary concept word] 10679

62 60 or 61 10679

63 mali.mp. or Mali/ 5366

64 mauritania.mp. or Mauritania/ 935

65 Mauritius/ 640

66 (mauritius or "agalega islands").mp. [mp=title, book title, abstract, original title, name of substance word, subject heading word, floating sub-heading word, keyword heading word, organism supplementary concept word, protocol supplementary concept word, rare disease supplementary concept word, unique identifier, synonyms, population supplementary concept word, anatomy supplementary concept word] 1365

67 65 or 66 1365

68 Mozambique/ 3233

69 (mozambique or "portugese east africa").mp. [mp=title, book title, abstract, original title, name of substance word, subject heading word, floating sub-heading word, keyword heading word, organism supplementary concept word, protocol supplementary concept word, rare disease supplementary concept word, unique identifier, synonyms, population supplementary concept word, anatomy supplementary concept word] 5405

70 68 or 69 5405

71 namibia.mp. or Namibia/ 2541

72 Niger/ or niger.mp. 17081

73 nigeria.mp. or Nigeria/ 49409

74 Rwanda/ 3399

75 (rwanda or ruanda).mp. [mp=title, book title, abstract, original title, name of substance word, subject heading word, floating sub-heading word, keyword heading word, organism supplementary concept word, protocol supplementary concept word, rare disease supplementary concept word, unique identifier, synonyms, population supplementary concept word, anatomy supplementary concept word] 5268

76 74 or 75 5268

77 "sao tome and principe".mp. or "Sao Tome and Principe"/ 234

78 senegal.mp. or Senegal/ 9092

79 seychelles.mp. or Seychelles/ 985

80 sierra leone.mp. or Sierra Leone/ 3459

81 somalia.mp. or Somalia/ 3139

82 south africa.mp. or South Africa/ 69803

83 south sudan.mp. or South Sudan/ 923

84 Sudan/ or sudan.mp. 11869

85 Tanzania/ 14734

86 (tanzania or tanganyika or zanzibar).mp. [mp=title, book title, abstract, original title, name of substance word, subject heading word, floating sub-heading word, keyword heading word, organism supplementary concept word, protocol supplementary concept word, rare disease supplementary concept word, unique identifier, synonyms, population supplementary concept word, anatomy supplementary concept word] 21221

87 85 or 86 21221

88 Togo/ 1333

89 (togo or "togolese republic").mp. [mp=title, book title, abstract, original title, name of substance word, subject heading word, floating sub-heading word, keyword heading word, organism supplementary concept word, protocol supplementary concept word, rare disease supplementary concept word, unique identifier, synonyms, population supplementary concept word, anatomy supplementary concept word] 2187

90 88 or 89 2187

91 uganda.mp. or Uganda/ 23809

92 Zambia/ 5832

93 (zambia or "northern rhodesia").mp. [mp=title, book title, abstract, original title, name of substance word, subject heading word, floating sub-heading word, keyword heading word, organism supplementary concept word, protocol supplementary concept word, rare disease supplementary concept word, unique identifier, synonyms, population supplementary concept word, anatomy supplementary concept word] 8480

94 92 or 93 8480

95 Zimbabwe/ 6795

96 (zimbabwe or rhodesia).mp. [mp=title, book title, abstract, original title, name of substance word, subject heading word, floating sub-heading word, keyword heading word, organism supplementary concept word, protocol supplementary concept word, rare disease supplementary concept word, unique identifier, synonyms, population supplementary concept word, anatomy supplementary concept word] 9615

97 95 or 96 9615

98 3 or 4 or 7 or 10 or 13 or 14 or 17 or 18 or 21 or 22 or 25 or 28 or 31 or 34 or 37 or 38 or 41 or 42 or 45 or 46 or 49 or 50 or 51 or 52 or 55 or 56 or 59 or 62 or 63 or 64 or 67 or 70 or 71 or 72 or 73 or 76 or 77 or 78 or 79 or 80 or 81 or 82 or 83 or 84 or 87 or 90 or 91 or 94 or 97 572919

99 ("metabolic disease*" or "cardiometabolic disease*").mp. [mp=title, book title, abstract, original title, name of substance word, subject heading word, floating sub-heading word, keyword heading word, organism supplementary concept word, protocol supplementary concept word, rare disease supplementary concept word, unique identifier, synonyms, population supplementary concept word, anatomy supplementary concept word] 49333

100 Cardiovascular Diseases/ 187796

101 "cardiovascular disease*".mp. [mp=title, book title, abstract, original title, name of substance word, subject heading word, floating sub-heading word, keyword heading word, organism supplementary concept word, protocol supplementary concept word, rare disease supplementary concept word, unique identifier, synonyms, population supplementary concept word, anatomy supplementary concept word] 353028

102 Noncommunicable Diseases/ 3728

103 ("non$communicable disease*" or "chronic disease*" or "non$infectious disease*").mp. [mp=title, book title, abstract, original title, name of substance word, subject heading word, floating sub-heading word, keyword heading word, organism supplementary concept word, protocol supplementary concept word, rare disease supplementary concept word, unique identifier, synonyms, population supplementary concept word, anatomy supplementary concept word] 370914

104 multimorbidity.mp. or Multimorbidity/ 9914

105 Chronic Disease/ 287829

106 ("multiple long term conditions" or "multiple long-term conditions").mp. [mp=title, book title, abstract, original title, name of substance word, subject heading word, floating sub-heading word, keyword heading word, organism supplementary concept word, protocol supplementary concept word, rare disease supplementary concept word, unique identifier, synonyms, population supplementary concept word, anatomy supplementary concept word] 221

107 Stroke/ 142103

108 (stroke or "cerebrovascular accident" or "vascular accident").mp. [mp=title, book title, abstract, original title, name of substance word, subject heading word, floating sub-heading word, keyword heading word, organism supplementary concept word, protocol supplementary concept word, rare disease supplementary concept word, unique identifier, synonyms, population supplementary concept word, anatomy supplementary concept word] 407390

109 Diabetes Mellitus/ 146376

110 (diabetes or diabetic).mp. [mp=title, book title, abstract, original title, name of substance word, subject heading word, floating sub-heading word, keyword heading word, organism supplementary concept word, protocol supplementary concept word, rare disease supplementary concept word, unique identifier, synonyms, population supplementary concept word, anatomy supplementary concept word] 898191

111 Insulin Resistance/ 70642

112 ("insulin resistance" or "insulin sensitivity").mp. [mp=title, book title, abstract, original title, name of substance word, subject heading word, floating sub-heading word, keyword heading word, organism supplementary concept word, protocol supplementary concept word, rare disease supplementary concept word, unique identifier, synonyms, population supplementary concept word, anatomy supplementary concept word] 136409

113 Non-alcoholic Fatty Liver Disease/ 27532

114 ("non-alcoholic fatty liver disease" or "nonalcoholic fatty liver disease" or "fatty liver" or steatohepatitis or nafld).mp. [mp=title, book title, abstract, original title, name of substance word, subject heading word, floating sub-heading word, keyword heading word, organism supplementary concept word, protocol supplementary concept word, rare disease supplementary concept word, unique identifier, synonyms, population supplementary concept word, anatomy supplementary concept word] 72868

115 Renal Insufficiency, Chronic/ 40516

116 ("chronic kidney disease*" or "chronic renal disease" or "chronic renal insufficienc*" or egfr or "glomerular filtration rate").mp. [mp=title, book title, abstract, original title, name of substance word, subject heading word, floating sub-heading word, keyword heading word, organism supplementary concept word, protocol supplementary concept word, rare disease supplementary concept word, unique identifier, synonyms, population supplementary concept word, anatomy supplementary concept word] 223263

117 Hypertension/ 265832

118 (hypertens* or "high blood pressure*").mp. [mp=title, book title, abstract, original title, name of substance word, subject heading word, floating sub-heading word, keyword heading word, organism supplementary concept word, protocol supplementary concept word, rare disease supplementary concept word, unique identifier, synonyms, population supplementary concept word, anatomy supplementary concept word] 628714

119 Dyslipidemias/ 15220

120 dyslipid$emia*.mp. [mp=title, book title, abstract, original title, name of substance word, subject heading word, floating sub-heading word, keyword heading word, organism supplementary concept word, protocol supplementary concept word, rare disease supplementary concept word, unique identifier, synonyms, population supplementary concept word, anatomy supplementary concept word] 47878

121 Hyperlipidemias/ 29078

122 hyperlipid$emia*.mp. [mp=title, book title, abstract, original title, name of substance word, subject heading word, floating sub-heading word, keyword heading word, organism supplementary concept word, protocol supplementary concept word, rare disease supplementary concept word, unique identifier, synonyms, population supplementary concept word, anatomy supplementary concept word] 51197

123 Obesity/ or obesity.mp. 435582

124 overweight.mp. or Overweight/ 100738

125 Anxiety/ or anxiety.mp. 340738

126 Depression/ 162369

127 depressi*.mp. [mp=title, book title, abstract, original title, name of substance word, subject heading word, floating sub-heading word, keyword heading word, organism supplementary concept word, protocol supplementary concept word, rare disease supplementary concept word, unique identifier, synonyms, population supplementary concept word, anatomy supplementary concept word] 606693

128 99 or 100 or 101 or 102 or 103 or 104 or 105 or 106 or 107 or 108 or 109 or 110 or 111 or 112 or 113 or 114 or 115 or 116 or 117 or 118 or 119 or 120 or 121 or 122 or 123 or 124 or 125 or 126 or 127 3548551

129 Mortality/ 50396

130 mortalit*.mp. [mp=title, book title, abstract, original title, name of substance word, subject heading word, floating sub-heading word, keyword heading word, organism supplementary concept word, protocol supplementary concept word, rare disease supplementary concept word, unique identifier, synonyms, population supplementary concept word, anatomy supplementary concept word] 1505110

131 Stress, Psychological/ 138035

132 stress*.mp. [mp=title, book title, abstract, original title, name of substance word, subject heading word, floating sub-heading word, keyword heading word, organism supplementary concept word, protocol supplementary concept word, rare disease supplementary concept word, unique identifier, synonyms, population supplementary concept word, anatomy supplementary concept word] 1368798

133 Prevalence/ or prevalence.mp. 956764

134 incidence.mp. or Incidence/ 1098239

135 "disease severity".mp. [mp=title, book title, abstract, original title, name of substance word, subject heading word, floating sub-heading word, keyword heading word, organism supplementary concept word, protocol supplementary concept word, rare disease supplementary concept word, unique identifier, synonyms, population supplementary concept word, anatomy supplementary concept word] 61980

136 Disease Management/ 43672

137 "disease* management*".mp. [mp=title, book title, abstract, original title, name of substance word, subject heading word, floating sub-heading word, keyword heading word, organism supplementary concept word, protocol supplementary concept word, rare disease supplementary concept word, unique identifier, synonyms, population supplementary concept word, anatomy supplementary concept word] 66979

138 Self-Management/ 6254

139 ("self management" or "self-management").mp. [mp=title, book title, abstract, original title, name of substance word, subject heading word, floating sub-heading word, keyword heading word, organism supplementary concept word, protocol supplementary concept word, rare disease supplementary concept word, unique identifier, synonyms, population supplementary concept word, anatomy supplementary concept word] 30856

140 "Quality of Life"/ 294649

141 (qol or hrqol or "quality of life" or "life quality").mp. [mp=title, book title, abstract, original title, name of substance word, subject heading word, floating sub-heading word, keyword heading word, organism supplementary concept word, protocol supplementary concept word, rare disease supplementary concept word, unique identifier, synonyms, population supplementary concept word, anatomy supplementary concept word] 507094

142 129 or 130 or 131 or 132 or 133 or 134 or 135 or 136 or 137 or 138 or 139 or 140 or 141 4928096

143 "Health Services Needs and Demand"/ 55760

144 (health* adj3 need*).mp. [mp=title, book title, abstract, original title, name of substance word, subject heading word, floating sub-heading word, keyword heading word, organism supplementary concept word, protocol supplementary concept word, rare disease supplementary concept word, unique identifier, synonyms, population supplementary concept word, anatomy supplementary concept word] 121884

145 "Delivery of Health Care"/ or Health Services Accessibility/ 207381

146 (health* adj3 barrier*).mp. [mp=title, book title, abstract, original title, name of substance word, subject heading word, floating sub-heading word, keyword heading word, organism supplementary concept word, protocol supplementary concept word, rare disease supplementary concept word, unique identifier, synonyms, population supplementary concept word, anatomy supplementary concept word] 13224

147 (health* adj3 facilitator*).mp. [mp=title, book title, abstract, original title, name of substance word, subject heading word, floating sub-heading word, keyword heading word, organism supplementary concept word, protocol supplementary concept word, rare disease supplementary concept word, unique identifier, synonyms, population supplementary concept word, anatomy supplementary concept word] 1394

148 143 or 144 or 145 or 146 or 147 320696

149 98 and 128 38028

150 142 or 148 5185851

151 149 and 150 18627

152 limit 151 to yr="2000 -Current" 16969

153 Infections/ 40933

154 infection*.mp. [mp=title, book title, abstract, original title, name of substance word, subject heading word, floating sub-heading word, keyword heading word, organism supplementary concept word, protocol supplementary concept word, rare disease supplementary concept word, unique identifier, synonyms, population supplementary concept word, anatomy supplementary concept word] 2464181

155 Neoplasms/ 529449

156 cancer*.mp. [mp=title, book title, abstract, original title, name of substance word, subject heading word, floating sub-heading word, keyword heading word, organism supplementary concept word, protocol supplementary concept word, rare disease supplementary concept word, unique identifier, synonyms, population supplementary concept word, anatomy supplementary concept word] 2475868

157 HIV/ 22700

158 Acquired Immunodeficiency Syndrome/ 79400

159 ("immune deficiency" or HIV or AIDS or immunodeficiency or leukemia or lymphadenopathy).mp. [mp=title, book title, abstract, original title, name of substance word, subject heading word, floating sub-heading word, keyword heading word, organism supplementary concept word, protocol supplementary concept word, rare disease supplementary concept word, unique identifier, synonyms, population supplementary concept word, anatomy supplementary concept word] 953437

160 malaria.mp. or Malaria/ 110799

161 153 or 154 or 155 or 156 or 157 or 158 or 159 or 160 5581875

162 152 not 161 12451

163 limit 162 to english language 12058

164        limit 163 to dt=20211201-20240122 [December 1st, 2021 to January 22nd, 2024]   2398

**Figure S1. Prevalence of hypertension**

**Figure S2. Prevalence of type 2 diabetes**


**Figure S3. Prevalence of hypercholesterolemia**

**Figure S4. Prevalence of cardiovascular disease**

**Figure S5. Prevalence of stroke**

**Table S1. Sub-Saharan African sub-regions and countries included in analysis**

| 1. **Central region** | 1. **Eastern region** | 1. **Northern region** | 1. **Southern region** | 1. **Western region** |
| --- | --- | --- | --- | --- |
| Angola | Ethiopia | Sudan | Botswana | Benin |
| Cameroon | Comoros |  | Lesotho | Bissau |
| Republic of Congo | Eritrea |  | Namibia | Burkina Faso |
|  | Kenya |  | South Africa | Benin |
|  | Tanzania |  | Zambia | Mali |
|  | Uganda |  |  | Liberia |
|  | Madagascar |  |  | Cabo Verde |
|  | Malawi |  |  | Côte d’Ivoire |
|  | Mauritius |  |  | Gabo |
|  | Mozambique |  |  | Ghana |
|  | Rwanda |  |  | Guinea |
|  | Somalia |  |  | Nigeria |
|  |  |  |  | Senegal |
|  |  |  |  | Sierra Leone |
|  |  |  |  | Gambia |
